# Supplementary material for: Membrane Domain Localization and Interaction of the Prion-Family Proteins, Prion and Shadoo with Calnexin
Source: Membranes (Basel). 2021 Dec 13;11(12):978. doi: 10.3390/membranes11120978 (PMC8704586; doi:10.3390/membranes11120978)
Supplement: Supplementary file 1 [file membranes-11-00978-s001.zip › membranes-1477933-supplementary.pdf]

SUPPLEMENTAL INFORMATION

Membrane domain localization and interaction of prion-family proteins prion and Shadoo with calnexin

Divya Teja Dondapati[1,2], Pradeep Reddy Cingaram[1,†], Ferhan Ayaydin[3,4], Antal Nyeste[5,6], Andor Kanyó[7,‡], Ervin Welker[1,6], Elfrieda Fodor[1]

[1] Institute of Biochemistry, Biological Research Centre, Szeged, Hungary.

[2] Doctoral School of Multidisciplinary Medical Sciences, University of Szeged, Szeged, Hungary.

[3] Hungarian Centre of Excellence for Molecular Medicine (HCEMM) Nonprofit Ltd., Szeged, Hungary.

[4] Cellular Imaging Laboratory, Biological Research Centre, Szeged, Hungary.

[5] Proteoscientia Ltd., Cserhátszentiván, Hungary.

[6] Institute of Enzymology, Research Centre for Natural Sciences, Budapest, Hungary.

[7] Biospirál-2006. Ltd., Szeged, Hungary.

† **Present address:** Pradeep Reddy Cingaram, Department of Human Genetics, Sylvester Comprehensive Cancer Center, University of Miami Miller School of Medicine, Miami, FL 33136, USA.

‡ **Present address:** Andor Kanyó, Department of Microbiology, Faculty of Science and Informatics, University of Szeged, Szeged, Hungary.

Supplemental Figure S1

(A)

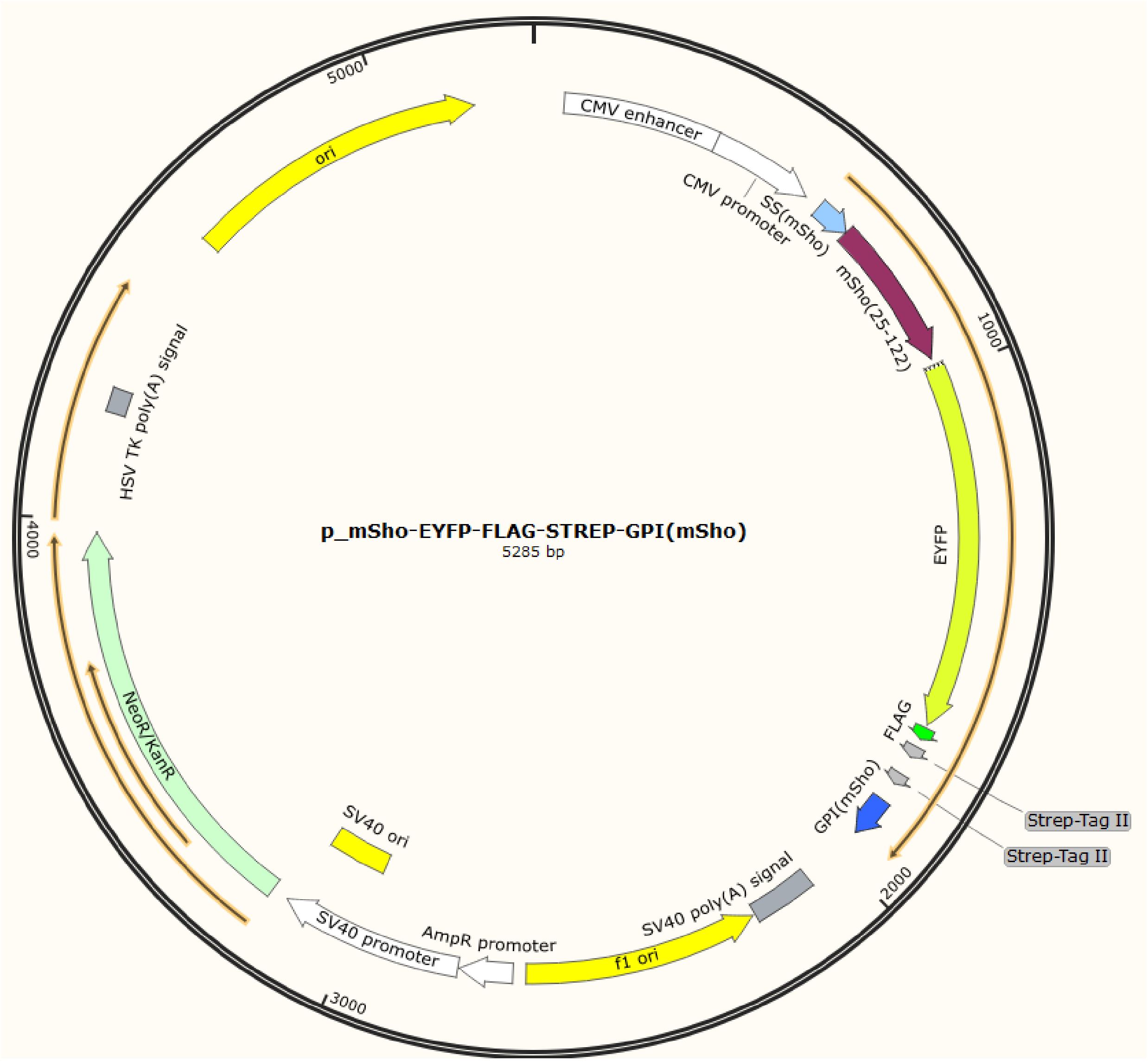

(B)

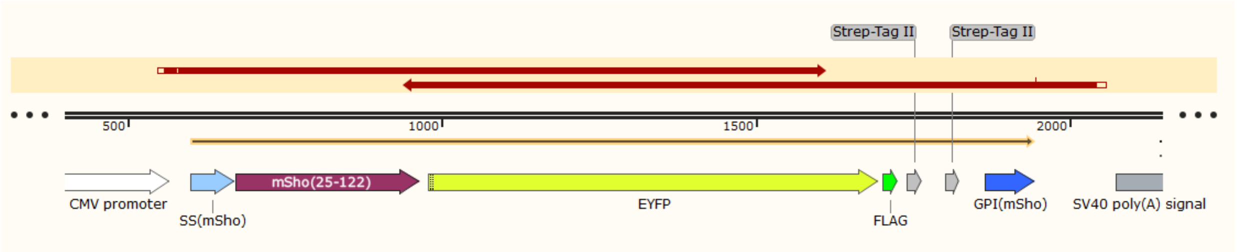

TAGCAGAGCTGGTTTAGTGACCGTCAGATCCGCTAGCCACCATGAACTGGACTGCTGCCACGTGCTGGGCTCTGCTGCTGGCC
GCCGCCTTCCTCTGTGACAGCTGTTCCGCCAAGGGCGGCCGCGGAGGCGCTCGAGGCAGTGCCCGGGGGGTGCGCGGAGGT
GCACGCGGGGCATCAAGAGTACGCGTAAGGCCGGCGCCCCGCTACGGCTCCTCTCTGCGCGTGGCGGCTGCAGGGGCAGCA

GCAGGGGCTGCAGCGGGTGTGGCTGCGGGCCTTGCTACCGGCTCTGGCTGGAGGAGGACCTCAGGGCCTGGAGAGCTAGGC
CTGGAGGACGATGAGAATGGGGCAATGGGAGGCAACGGAACCGACCGAGGAGTCTACAGCTACTGGGCCTGGACTTCCTTA
CCGGTCGCCCATATGGTGAGCAAGGGCGAGGAGCTGTTCACCGGGGTGGTGCCCATCCTGGTCGAGCTGGACGGCGACGTA
AACGGCCACAAGTTCAGCGTGTCCGGCGAGGGCGAGGGCGATGCCACCTACGGCAAGCTGACCCTGAAGTTCATCTGCACCA
CCGGCAAGCTGCCCGTGCCCTGGCCCACCCTCGTGACCACCTTCGGCTACGGCCTGCAGTGCTTCGCCCGCTACCCCGACCACA
TGAAGCAGCACGACTTCTTCAAGTCCGCCATGCCCGAAGGCTACGTCCAGGAGCGCACCATCTTCTTCAAGGACGACGGCAAC
TACAAGACCCGCGCCGAGGTGAAGTTCGAGGGCGACACCCTGGTGAACCGCATCGAGCTGAAGGGCATCGACTTCAAGGAG
GACGGCAACATCCTGGGGCACAAGCTGGAGTACAACTACAACAGCCACAACGTCTATATCATGGCCGACAAGCAGAAGAACG
GCATCAAGGTGAACTTCAAGATCCGCCACAACATCGAGGACGGCAGCGTGCAGCTCGCCGACCACTACCAGCAGAACACCCCC
ATCGGCGACGGCCCCGTGCTGCTGCCCGACAACCACTACCTGAGCTACCAGTCCGCCCTGAGCAAAGACCCCAACGAGAAGC
GCGATCACATGGTCCTTAAGGAGTTCGTGACCGCCGCCGGGATCACTCTCGGCATGGACGAGCTGTACAAGATGCAGGATTAT
AAAGATGATGATGATAAAGGGTCGGCCGCCAGCTGGAGCCACCCTCAGTTCGAGAAGGGAGGAGGAAGCGGCGGAGGCAG
CGGAGGAGGAAGCTGGAGCCACCCGCAGTTCGAGAAAGGAGCTAGATCAAGATCTCGAGCTCAAGCTTCGAATTCTGGCTCA
GGGTCCGTGCACAGCCCACGTATTTGTCTGCTTCTGGGCGGCACCCTTGGTGCCCTAGAACTGCTTCGGCCTTAGGGATCCACC
GGATCTAGATAACTGATCATAATCAGCCATACCACATTTGTAGAGGTTTTACTTGCTTTAAAAAACCTCCCACACCTCCCCCTGA
ACCNAAACATAAAANNNNNNNT

____: SS(mSho) ____: mSho(25-122); ____: EYFP; ____: FLAG-tag; ____: Strep-tag II; ____: GPI(mSho)-signal peptide

Supplemental Figure S1. Map of the plasmid DNA **p_mSho-EYFP-FLAG-GPI(mSho)**, used for generating the N2a/Sho-EYFP-FLAG cells. The plasmid encodes for the expression of mouse Shadoo (mSho) protein in fusion with EYFP-, FLAG- and Strep-tag II tags at its C-terminal, upstream of its GPI-signal peptide sequence. A, Circular map of the plasmid, indicating the major features along the DNA sequence, with the top three open reading frames (thin orange arrows). B, Horizontal map of the region of Sanger-sequenced region (indicated by the top, red-coloured forward and reverse arrows) comprising the coding region of the fusion protein constructed. The translated region is indicated by thin orange arrow, below which the major features are indicated on the map. The resulting sequence from Sanger-sequencing is shown below the map, with the specific features highlighted by colors, as indicated, encoding for as follows: secretion signal of mSho, SS(mSho) (cyan); mature mShadoo protein, mSho(25-122) (pink); EYFP (yellow); FLAG-tag (green); two Strep-tag II tags (grey) and the sequence of GPI-anchor signal peptide of mSho, GPI(mSho)-signal peptide (blue).

Supplemental Figure S2

(A)

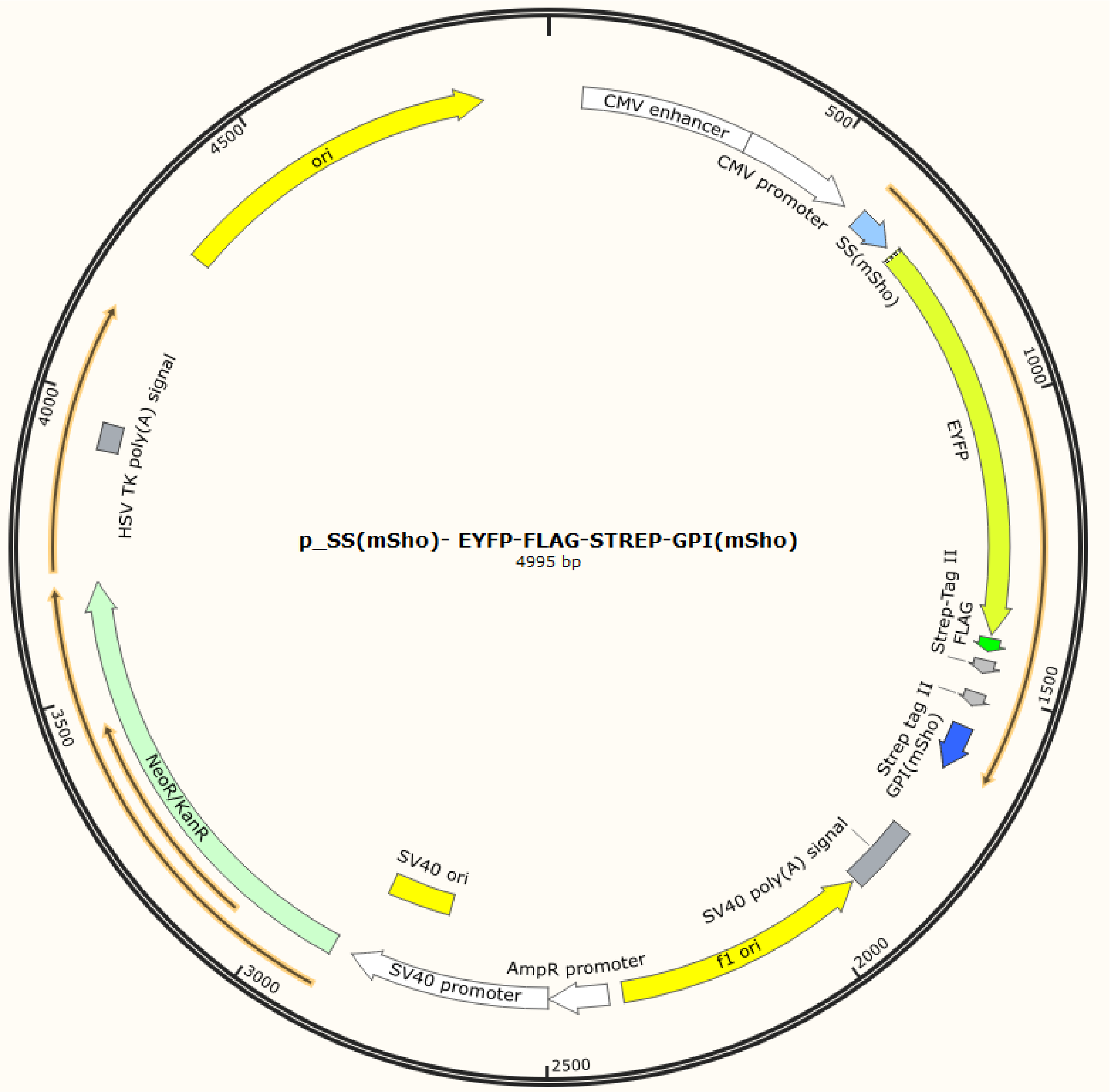

(B)

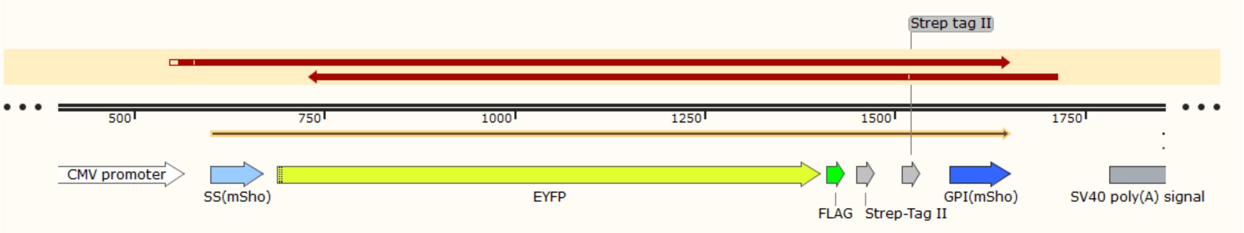

TNNNTGNNNNYNNTAGCAGAGCTGGTTTAGTGACCGTCAGATCCGCTAGCCACCATGAACTGGACTGCTGCCACGTGCTGGG
CTCTGCTGCTGGCCGCCGCCTTCCTCTGTGACAGCTGTAGCGCCTTACCGGTCGCCCATATGGTGAGCAAGGGCGAGGAGCTG
TTCACCGGGGTGGTGCCCATCCTGGTCGAGCTGGACGGCGACGTAAACGGCCACAAGTTCAGCGTGTCCGGCGAGGGCGAG
GGCGATGCCACCTACGGCAAGCTGACCCTGAAGTTCATCTGCACCACCGGCAAGCTGCCCGTGCCCTGGCCCACCCTCGTGAC
CACCTTCGGCTACGGCCTGCAGTGCTTCGCCCGCTACCCCGACCACATGAAGCAGCACGACTTCTTCAAGTCCGCCATGCCCGA
AGGCTACGTCCAGGAGCGCACCATCTTCTTCAAGGACGACGGCAACTACAAGACCCGCGCCGAGGTGAAGTTCGAGGGCGAC
ACCCTGGTGAACCGCATCGAGCTGAAGGGCATCGACTTCAAGGAGGACGGCAACATCCTGGGGCACAAGCTGGAGTACAACT
ACAACAGCCACAACGTCTATATCATGGCCGACAAGCAGAAGAACGGCATCAAGGTGAACTTCAAGATCCGCCACAACATCGA
GGACGGCAGCGTGCAGCTCGCCGACCACTACCAGCAGAACACCCCCATCGGCGACGGCCCCGTGCTGCTGCCCGACAACCAC
TACCTGAGCTACCAGTCCGCCCTGAGCAAAGACCCCAACGAGAAGCGCGATCACATGGTCCTTAAGGAGTTCGTGACCGCCGC
CGGGATCACTCTCGGCATGGACGAGCTGTACAAGATGCAGGATTATAAAGATGATGATGATAAAGGGTCGGCCGCCAGCTGG
AGCCACCCTCAGTTCGAGAAGGGAGGAGGAAGCGGCGGAGGCAGCGGAGGAGGAAGCTGGAGCCACTCGCAGTTCGAGAA
AGGAGCTAGATCAAGATCTCGAGCTCAAGCTTCGAATTCTGGCTCAGGGTCCGTGCACAGCCCACGTATTTGTCTGCTTCTGGG
CGGCACCCTTGGTGCCCTAGAACTGCTTCGGCCTTAG

_____: SS(mSho); _____: EYFP; _____: FLAG-tag; _____: Strep-tag II; _____: GPI(mSho)-signal peptide

Supplemental Figure S2. Map of the plasmid DNA **p_SS(mSho)-EYFP-FLAG-GPI(mSho)**, used for generating the N2a/EYFP-FLAG cells. The plasmid is constructed to express the control protein EYFP with the ER signal sequence of mSho and in fusion with a FLAG- and two Strep-tag II tags followed by the GPI-signal peptide of mSho, respectively, at its C-terminus. A, Circular map of the plasmid, indicating the major features along the DNA sequence, with the top three open reading frames marked (thin orange arrows). B, Horizontal map of sequences region containing the Sanger-sequenced segments (indicated by the top, red-coloured forward and reverse arrows) comprising the coding region of the fusion protein constructed. The translated region is indicated by thin orange arrow, below which the major features are indicated. The resulting sequence from Sanger-sequencing is shown below the map, with the specific features highlighted by colors, as indicated on the figure, encoding for as follows: secretion signal of mSho, SS(mSho) (cyan); EYFP (yellow); FLAG-tag (green); two Strep-tag II tags (gray) and the sequence of the signal peptide of GPI-anchor of mSho, GPI(mSho)-signal peptide (blue). Note: according to the sequencing, the second Strep-Tag II contains a point mutation (dark grey).

Supplemental Figure S3

(A)

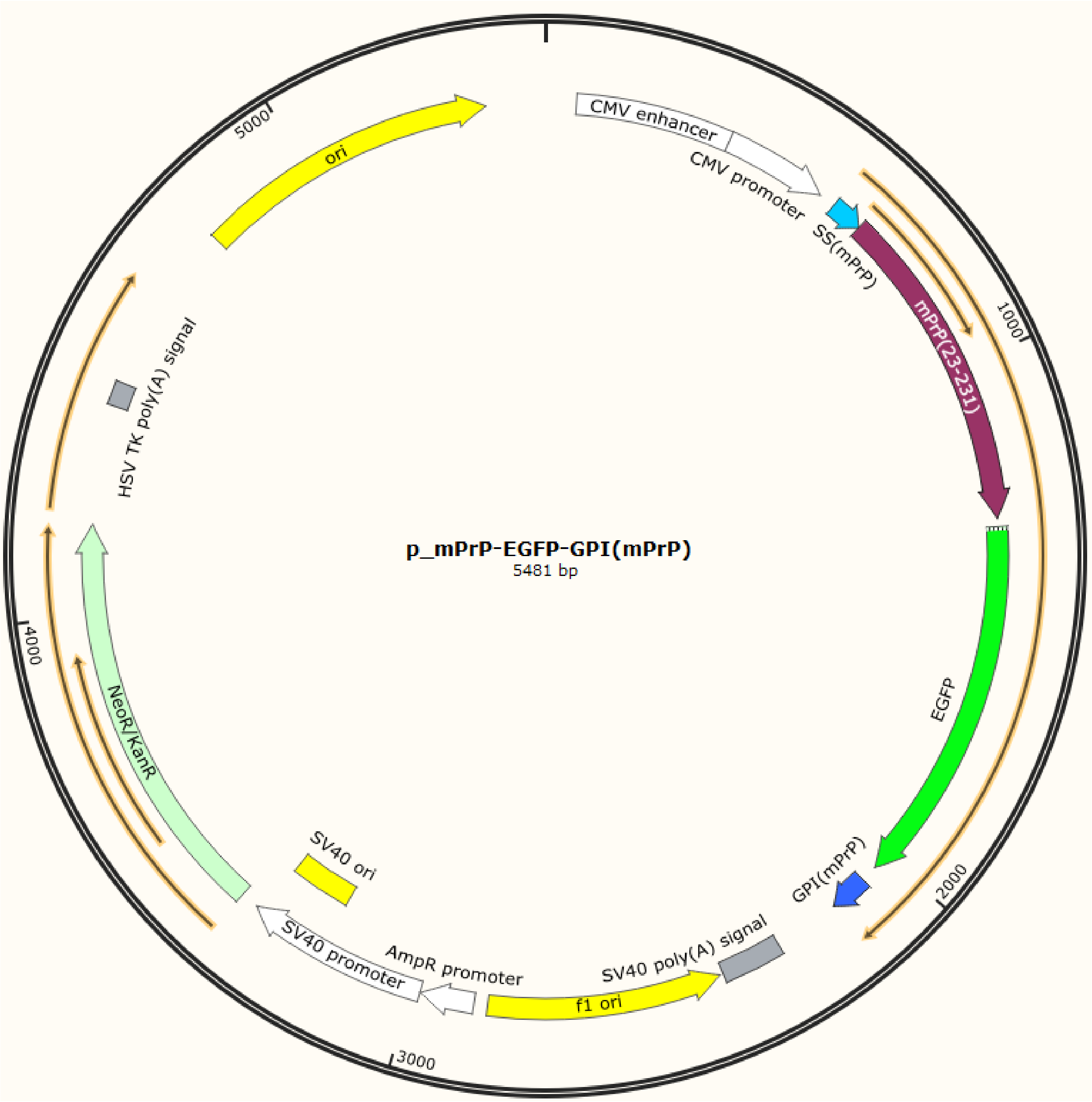

(B)

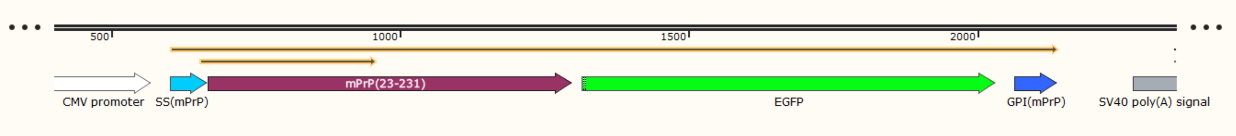

GCCAAGTACGCCCCCTATTGACGTCAATGACGGTAAATGGCCCGCCTGGCATTATGCCCAGTACATGACCTTATGGGACTTTCC
TACTTGGCAGTACATCTACGTATTAGTCATCGCTATTACCATGGTGATGCGGTTTTGGCAGTACATCAATGGGCGTGGATAGCG
GTTTGACTCACGGGGATTTCCAAGTCTCCACCCCATTGACGTCAATGGGAGTTTGTTTTGGCACCAAAATCAACGGGACTTTCC

AAAATGTCGTAACAACTCCGCCCCATTGACGCAAATGGGCGGTAGGCGTGTACGGTGGGAGGTCTATATAAGCAGAGCTGGT
TTAGTGAACCGTCAGATCCGCTAGCCACCATGGCGAACCTTGGCTACTGGCTGCTGGCCCTCTTTGTGACTATGTGGACTGATG
TCGGCCTCTGCAAAAAGCGGCCAAAGCCTGGAGGGTGGAACACCGGTGGAAGCCGGTATCCCGGGCAGGGAAGCCCTGGAG
GCAACCGTTACCCACCTCAGGGTGGCACCTGGGGGCAGCCCCACGGTGGTGGCTGGGGACAACCCCATGGGGGCAGCTGGG
GACAACCTCATGGTGGTAGTTGGGGTCAGCCCCATGGCGGTGGATGGGGCCAAGGAGGGGGTACCCATAATCAGTGGAACA
AGCCCAGCAAACCAAAAACCAACCTCAAGCATGTGGCAGGGGCTGCGGCAGCTGGGGCAGTAGTGGGGGGCCTTGGTGGCT
ACATGCTGGGGAGCGCCATGAGCAGGCCCATGATCCATTTTGGCAACGACTGGGAGGACCGCTACTACCGTGAAAACATGTA
CCGCTACCCTAACCAAGTGTACTACAGGCCAGTGGATCAGTACAGCAACCAGAACAACTTCGTGCACGACTGCGTCAATATCA
CCATCAAGCAGCACACGGTCACCACCACCACCAAGGGGGAGAACTTCACCGAGACCGATGTGAAGATGATGGAGCGCGTGGT
GGAGCAGATGTGCGTCACCCAGTACCAGAAGGAGTCCCAGGCCTATTACGACGGGAGAAGATCCAGCGGGCCCGGACCGGT
CGCCACCATGGTGAGCAAGGGCGAGGAGCTGTTCACCGGGGTGGTGCCCATCCTGGTCGAGCTGGACGGCGACGTAAACGG
CCACAAGTTCAGCGTGTCCGGCGAGGGCGAGGGCGATGCCACCTACGGCAAGCTGACCCTGAAGTTCATCTGCACCACCGGC
AAGCTGCCCGTGCCCTGGCCCACCCTCGTGACCACCCTGACCTACGGCGTGCAGTGCTTCAGCCGCTACCCCGACCACATGAA
GCAGCACGACTTCTTCAAGTCCGCCATGCCCGAAGGCTACGTCCAGGAGCGCACCATCTTCTTCAAGGACGACGGCAACTACA
AGACCCGCGCCGAGGTGAAGTTCGAGGGCGACACCCTGGTGAACCGCATCGAGCTGAAGGGCATCGACTTCAAGGAGGACG
GCAACATCCTGGGGCACAAGCTGGAGTACAACTACAACAGCCACAACGTCTATATCATGGCCGACAAGCAGAAGAACGGCAT
CAAGGTGAACTTCAAGATCCGCCACAACATCGAGGACGGCAGCGTGCAGCTCGCCGACCACTACCAGCAGAACACCCCCATCG
GCGACGGCCCCGTGCTGCTGCCCGACAACCACTACCTGAGCACCCAGTCCGCCCTGAGCAAAGACCCCAACGAGAAGCGCGA
TCACATGGTCCTGCTGGAGTTCGTGACCGCCGCCGGGATCACTCTCGGCATGGACGAGCTGTACAAGTCCGGACTCAGATCTC
GAGCTCAAGCTTCGAATTCTAGCAGCACCGTGCTTTTCTCCTCCCCTCCTGTCATCCTCCTCATCTCCTTCCTCATCTTCCTGATCG
TAGGCTAGCCCACCGGATCTAGATAACTGATCATAATCAGCCATACCACATTTGTAGAGGTTTTACTTGCTTTAAAAAACCTCCC
ACACCTCCCCCTGAACCTGAAACATAAAATGAATGCAATTGTTGTTGTTAACTTGTTTATTGCAGCTTATAATGGTTACAAATAA
AGCAATAGCATCACAAATTTCACAAATAAAGCATTTTTTTCACTGCATTCTAGTTGTGGTTTGTCCAAACTCATCAATGTATCTTA
ACGCGTAAATTGTAAGCGTTAATATTTTGTTAAAATTCGCGTTAAATTTTTGTTAAATCAGCTCATTTTTTAACCAATAGGCCGA
AATCGGCAAAATCCCTTATAAATCAAAAGAATAGACCGAGATAGGGTTGAGTGTTGTTCCAGT

_____: SS(mPrP), _____: mPrP(23-231); _____: EGFP; _____: GPI(mPrP)-signal peptide

Supplemental Figure S3. Map of the plasmid DNA **p_mPrP-EGFP-GPI(mPrP)**, used for generating the N2a/PrP-EGFP cells. The plasmid is constructed to express the mouse prion protein (mPrP) in fusion with EGFP at its C-terminus, upstream of its GPI-signal peptide. A, Circular map of the plasmid, indicating the major features along the DNA sequence, with the top three open reading frames marked (thin orange arrows). B, Horizontal map of the sequence region harbouring the fusion protein's coding sequence, with the major features indicated. The sequence of the plasmid-DNA segment comprising the coding region of the constructed fusion-protein is given below the map. The color-coded features encode for the protein-segments as indicated and as follows: the secretion signal of mPrP, SS(mPrP) (cyan); mature mPrP protein, mPrP(23-231) (pink); EGFP (green); sequence of the GPI-anchor signal peptide of mPrP, GPI(mPrP-signal peptide (blue).

Supplemental Figure S4

(A)

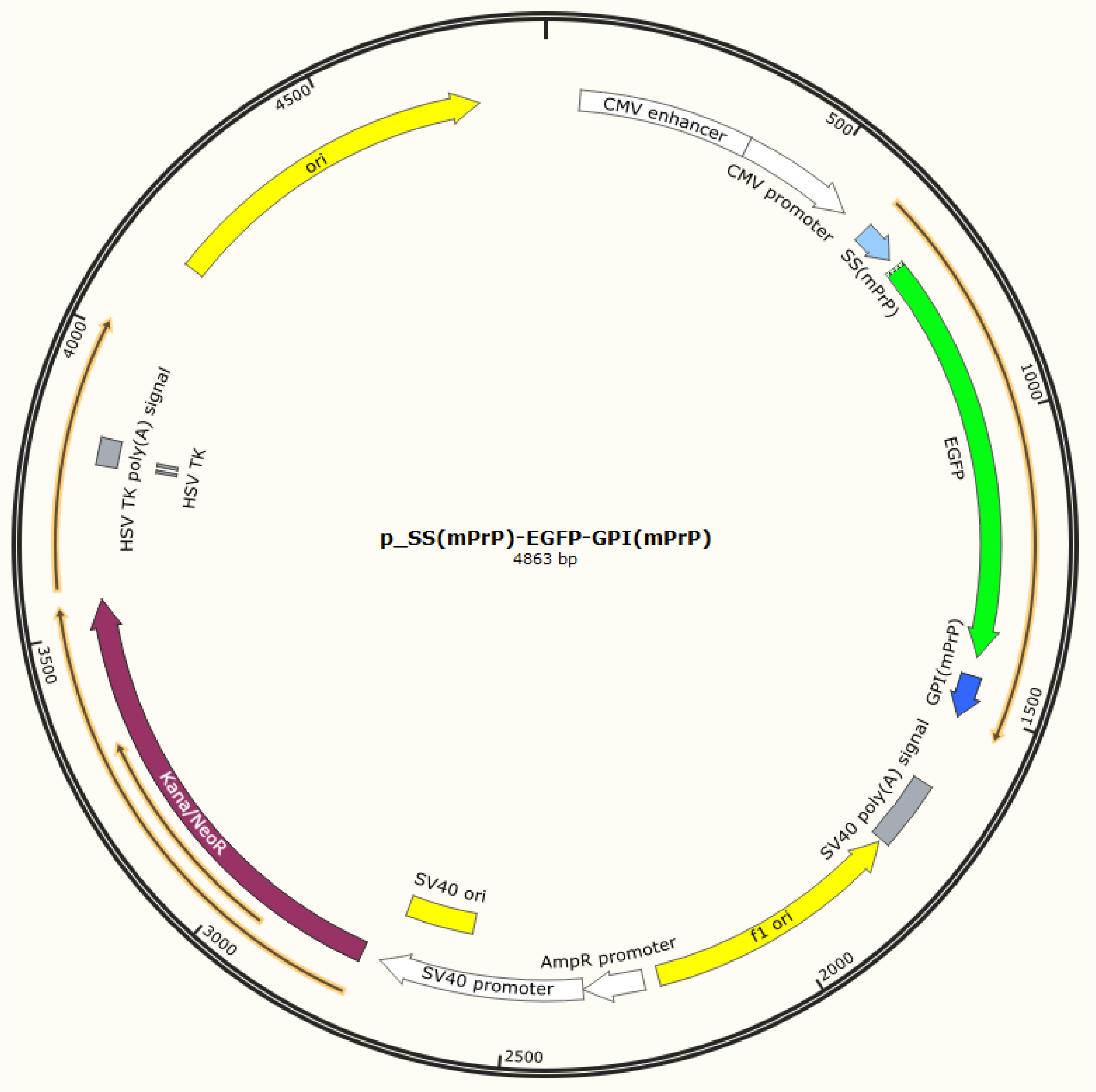

(B)

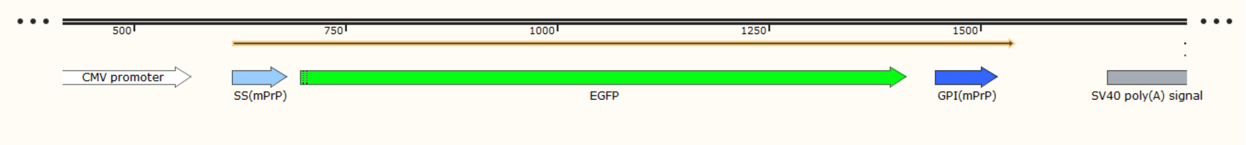

AGTCTCCACCCCATTGACGTCAATGGGAGTTTGTTTTGGCACCAAAATCAACGGGACTTTCCAAAATGTCGTAACA
ACTCCGCCCCATTGACGCAAATGGGCGGTAGGCGTGTACGGTGGGAGGTCTATATAAGCAGAGCTGGTTTAGTGA
ACCGTCAGATCCGCTAGCGCTACCGGTATCAGTCATCATGGCGAACCTTGGCTACTGGCTGCTGGCCCTCTTTGTG

ACTATGTGGACTGATGTAGGCCTCTGCTTACCGGTCGCCACCATGGTGAGCAAGGGCGAGGAGCTGTTCACCGGG
GTGGTGCCCATCCTGGTCGAGCTGGACGGCGACGTAAACGGCCACAAGTTCAGCGTGTCCGGCGAGGGCGAGGG
CGATGCCACCTACGGCAAGCTGACCCTGAAGTTCATCTGCACCACCGGCAAGCTGCCCGTGCCCTGGCCCACCCTC
GTGACCACCCTGACCTACGGCGTGCAGTGCTTCAGCCGCTACCCCGACCACATGAAGCAGCACGACTTCTTCAAGT
CCGCCATGCCCGAAGGCTACGTCCAGGAGCGCACCATCTTCTTCAAGGACGACGGCAACTACAAGACCCGCGCCG
AGGTGAAGTTCGAGGGCGACACCCTGGTGAACCGCATCGAGCTGAAGGGCATCGACTTCAAGGAGGACGGCAAC
ATCCTGGGGCACAAGCTGGAGTACAACTACAACAGCCACAACGTCTATATCATGGCCGACAAGCAGAAGAACGGC
ATCAAGGTGAACTTCAAGATCCGCCACAACATCGAGGACGGCAGCGTGCAGCTCGCCGACCACTACCAGCAGAAC
ACCCCCATCGGCGACGGCCCCGTGCTGCTGCCCGACAACCACTACCTGAGCACCCAGTCCGCCCTGAGCAAAGACC
CCAACGAGAAGCGCGATCACATGGTCCTGCTGGAGTTCGTGACCGCCGCCGGGATCACTCTCGGCATGGACGAGC
TGTACAAGTCCGGACTCAGATCTCGAGCTCAAGCTTCGAATTCTAGCAGCACCGTGCTTTTCTCCTCCCCTCCTGTC
ATCCTCCTCATCTCCTTCCTCATCTTCCTGATCGTGGGTGGATCCACCGGATCTAGATAACTGATCATAATCAGCCAT
ACCACATTTGTAGAGGTTTTACTTGCTTTAAAAAACCTCCCACACCTCCCCCTGAACCTGAAACATAAAATGAATGC
AATTGTTGTTGTTAACTTGTTTATTGCAGCTTATAATGGTTACAAATAAAGCAATAGCATCACAAATTTCACAAATA
A

: SS(mPrP); : EGFP; : GPI(mPrP)-signal peptide

Supplemental Figure S4. Map of the plasmid DNA **p_SS(mPrP)-EGFP-GPI(mPrP)**, used for generation of the N2a/EGFP cells. The plasmid is constructed to express the control fusion protein composed of EGFP in fusion with the ER signal peptide of mPrP at its N-terminus and with the GPI-signal peptide of mPrP at its C-terminus. A, Circular map of the plasmid, indicating the major features along the DNA sequence, with the top three open reading frames marked (thin orange arrows). B, Horizontal map of the region harbouring the fusion protein's coding sequence, with the major features indicated. The sequence of the plasmid-DNA segment comprising the coding region of the constructed fusion-protein is given below the map. The color-coded features encode for the protein-segments as indicated and as follows: the secretion signal of mPrP, SS(mPrP) (cyan); EGFP (green); the sequence of the GPI-anchor signal peptide of mPrP, GPI(mPrP)-signal peptide (blue).

Supplemental Figure S5

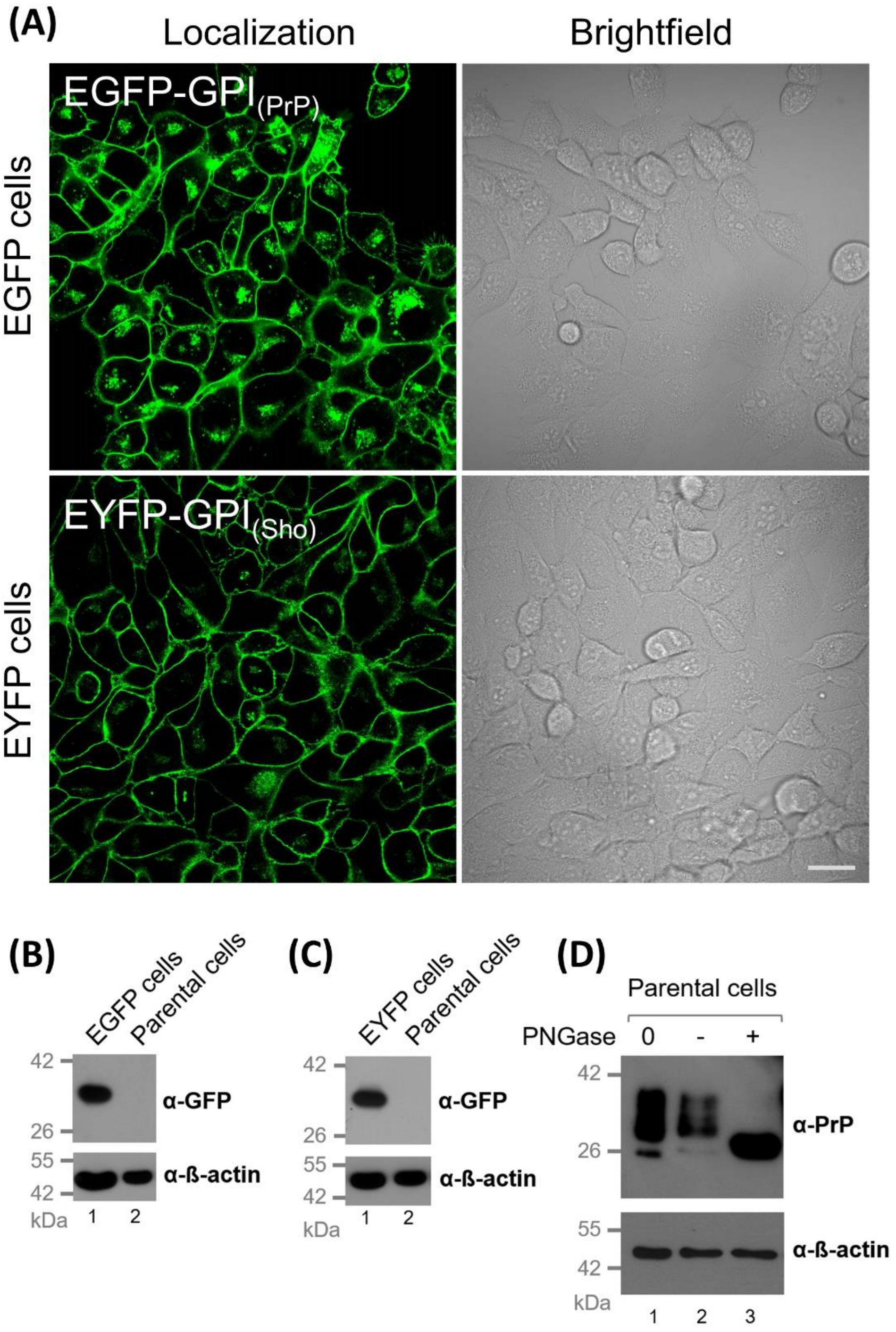

Supplemental Figure S5. Localization and expression levels of the control proteins in the control transgenic N2a cells. A, Representative live-cell confocal fluorescence and brightfield images of EGFP- (top panels) and EYFP- (bottom panels) cells, stably expressing the fluorescent proteins only equipped with the corresponding GPI-anchors: EGFP-$GPI_{(PrP)}$ and EYFP-$GPI_{(Sho)}$, made to serve as controls for PrP-EGFP- and Sho-EYFP cells, respectively. Images are acquired with 60x oil immersion objective and a Fluoview FV1000 confocal laser scanning microscope. Scale bar: 10 μm. B,C, Western blot analysis of the expressed transgenes in total cell lysates of EGFP- (B), and EYFP (C) cells. Anti-GFP antibody (α-GFP) is used to confirm the expression of the fluorescent proteins EGFP-$GPI_{(PrP)}$ (B) and EYFP-$GPI_{(Sho)}$ (C). β-actin is used as loading control and it is probed by anti- β-actin antibody (α-β-actin). D, Western blot analysis of complex N-glycosylation of endogenous PrP^C in the parental N2a cells. Untreated (0), treated in the absence of PNGase F (-) and PNGase F treated (+) total cell lysate samples of parental N2a cells are probed using anti-prion SAF-32 antibody (α-PrP) and shows that PrP^C is complex glycosylated. β-actin is used as loading control. M: molecular weight marker. All gels used are 12% polyacrylamide (PA) SDS gels.

Supplemental Figure S6

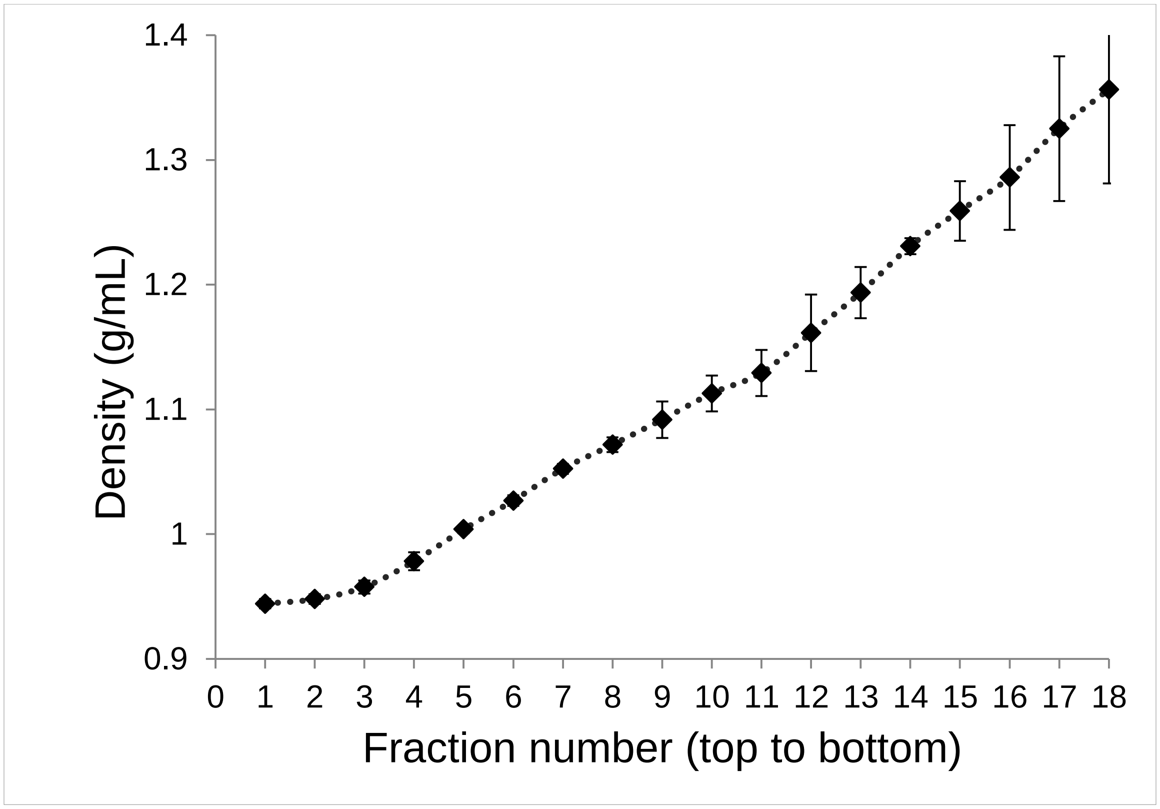

Supplemental Figure S6. Mean densities of the OptiPrep-Sucrose gradient fractions. To measure the characteristic densities of the fractions collected, control gradients are prepared without sample, briefly as follows. Buffer A with the enzymes, but without any sample is loaded at the bottom of the centrifuge tube and the continuous OptiPrep density-gradient is layered on top, similarly as for the samples, and the tubes are centrifuged in parallel with those containing the samples. Equal-volume-fractions are collected the same way from each tube in each experiment. Density values of fractions collected from the control-tubes are measured and calculated as described in Materials and Methods. Error bars represent the standard deviation of three independent gradients, made and processed during separate, independent experiments.

Supplemental Figure S7

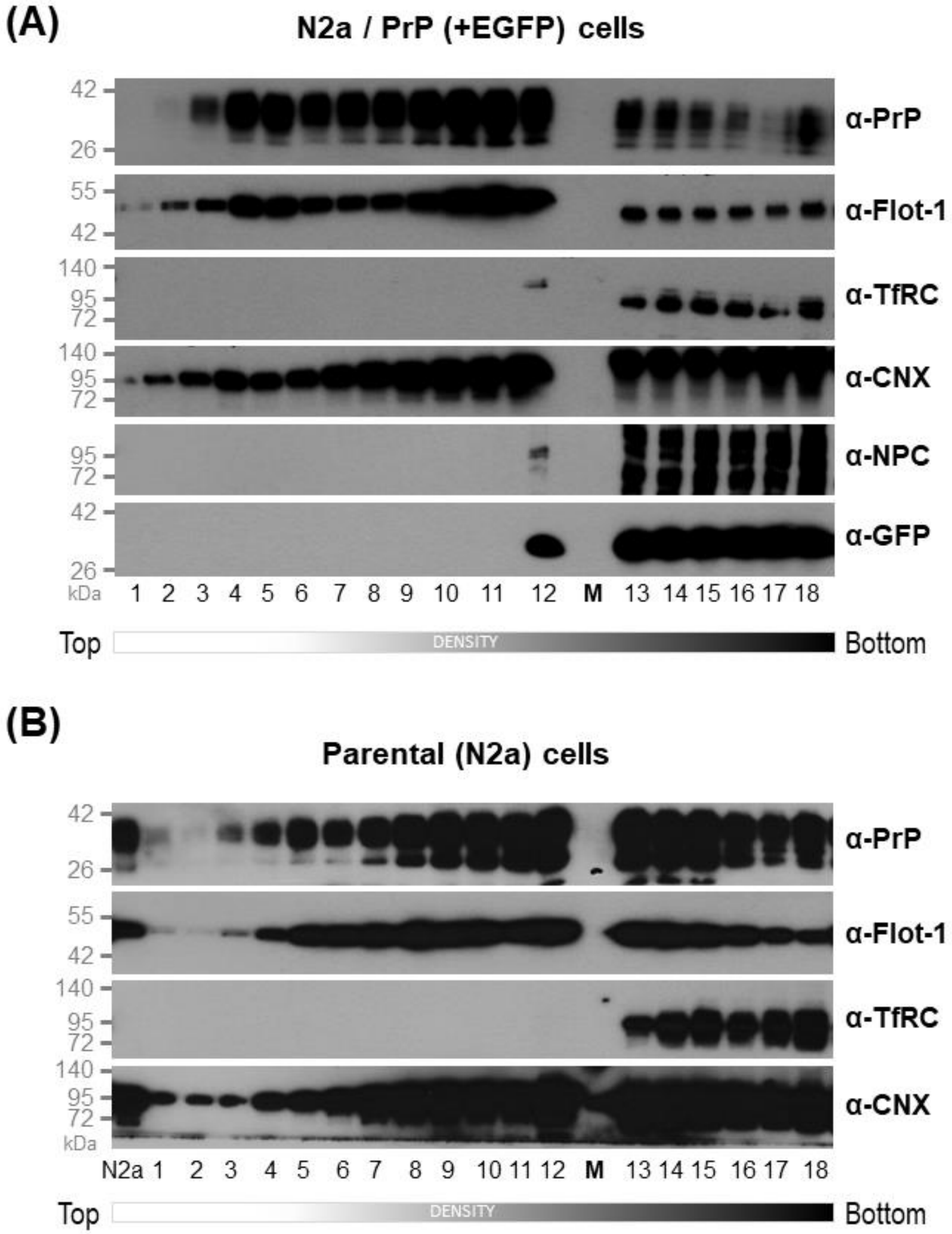

Supplemental Figure S7. Distribution of selected proteins across the OptiPrep density gradient fractions of prion overexpressing- and parental N2a cells. A, B, Transgenic N2a cells overexpressing prion protein without any fluorescent fusion tag and at the same time a soluble EGFP introduced on the same plasmid-DNA for transfection monitoring purposes, termed as N2a/PrP (+EGFP) cells (A) and parental N2a cells (B) are lysed and fractionated on OptiPrep density gradient. The distribution of selected proteins along the gradient fractions is tested by Western blotting with specific antibodies: SAF-32 antibody is used to detect the prion protein (α-PrP), antibodies α-Flotillin-1 (noted as "α-Flot-1"), α-TfRC, α-CNX, α-NPC are used to detect flotillin-1, transferrin receptor protein (TfRC), calnexin (CNX) and nuclear pore complex protein (NPC), respectively. M: protein molecular weight ladder. All gels are 12%- except for NPC which is 10% PA SDS gel.

Supplemental Figure S8

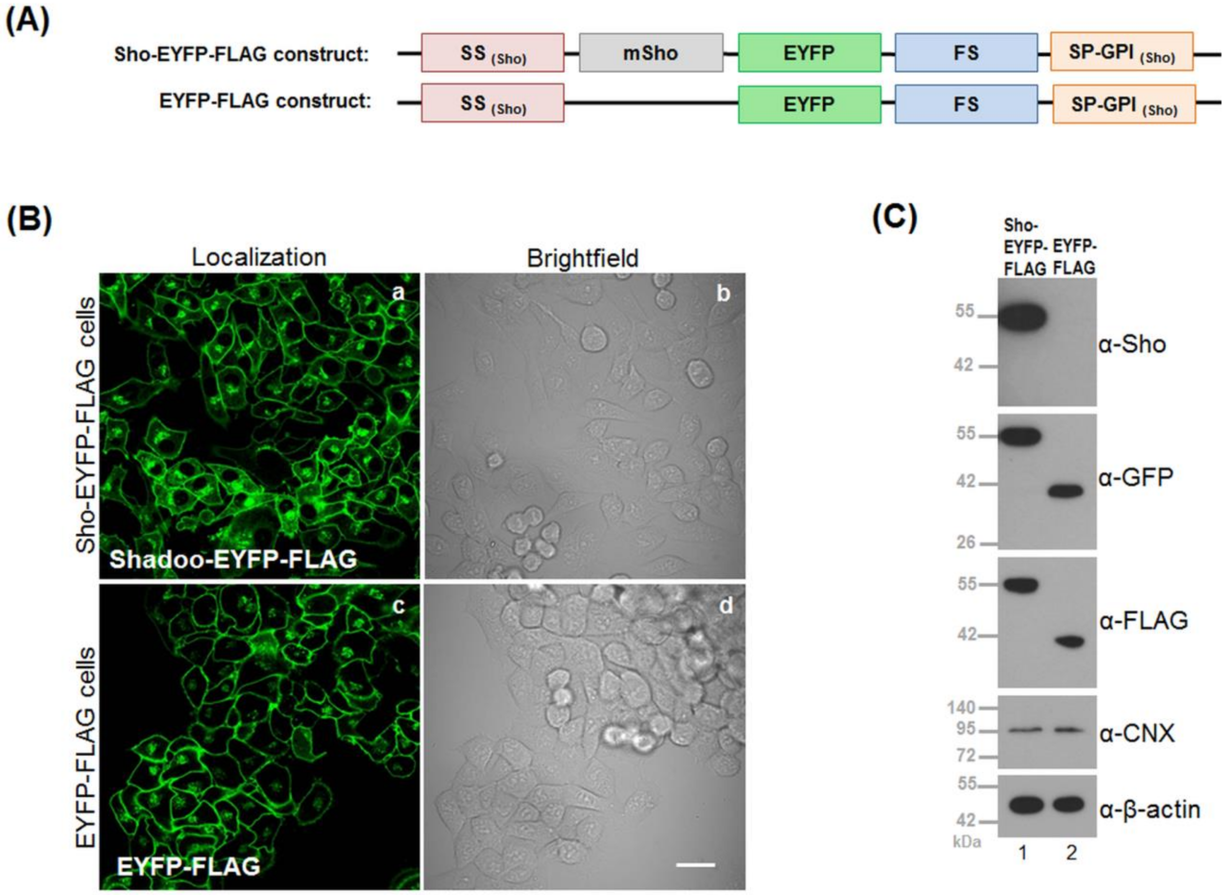

Supplemental Figure S8. Generation of the Sho-EYFP-FLAG- and EYFP-FLAG cells and the expression levels of the transgenes in the cells. A, Schematics of the DNA constructs used for developing the transgenic cells, encoding the FLAG-tagged Sho protein construct Sho-EYFP-FLAG-GPI$_{(Sho)}$ and the corresponding control protein EYFP-FLAG-GPI$_{(Sho)}$. The major sequence elements are depicted, coding for as follows. SS$_{(Sho)}$: N-terminal signal peptide of Sho; mSho: mouse Shadoo protein; EYFP: yellow fluorescent protein, FS: FLAG-STREPTAVIDIN-tag sequence; GPI$_{(Sho)}$: GPI-anchor attachment signal sequence of Sho. Note: only FLAG-tag is used in the experiments presented, therefore, in the naming of cells and proteins only FLAG-tag is mentioned. B, Representative live-cell confocal fluorescence and brightfield images of the stable transgenic Sho-EYFP-FLAG- (top panels) and its control, EYFP-FLAG (bottom panels) cells, made by transfecting N2a cells with plasmids encoding for the protein constructs on (A). The localization of the fluorescent fusion proteins in the stable cells is imaged using 60x oil

immersion objective of confocal laser scanning microscope Olympus Fluoview FV1000. Signals from EYFP are shown in green. Scale bar: 10 μm. C, Expression of transgenes analyzed by Western blotting of the total cell lysates of Sho-EYFP-FLAG and the EYFP-FLAG cells, as indicated on the figure, probing for Shadoo (α-Sho), EYFP (α-GFP), FLAG (α-FLAG) and calnexin (α-CNX). β-actin is used as loading control and it is probed by α-β-actin antibody.

Supplemental Figure S9

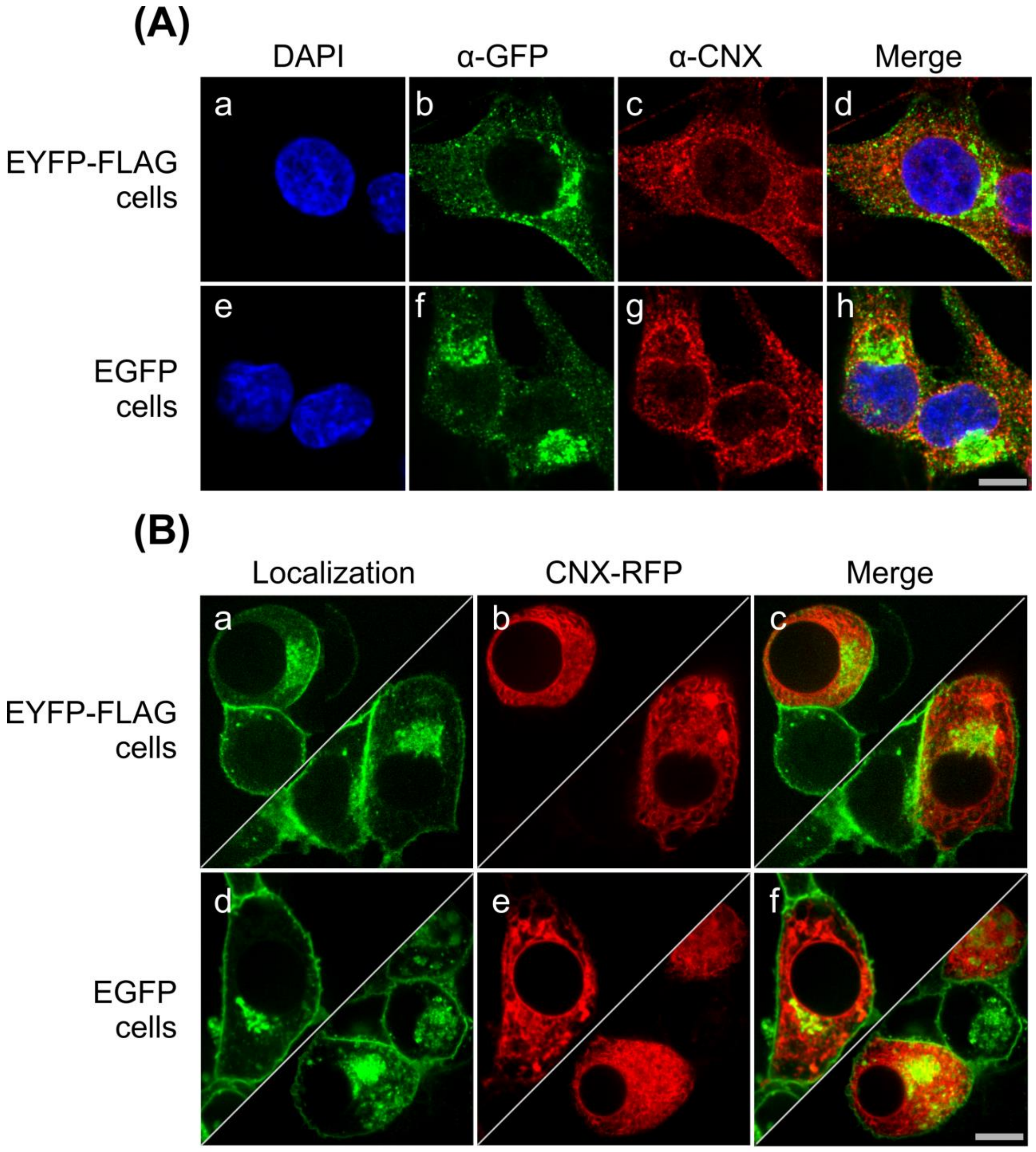

Supplemental Figure S9. Localization of the control proteins, EYFP-FLAG-GPI$_{(Sho)}$ and EGFP-GPI$_{(PrP)}$ and calnexin in the stable-transgenic EYFP-FLAG- and EGFP cells. A,

Immunocytochemistry and confocal microscopy images of EYFP-FLAG (a-d) and EGFP (e-h) cells. Cells are fixed, permeabilized and immunoprobed for the expressed proteins using α-GFP primary antibody and Alexa Fluor 488-labelled secondary antibody (b, f: green color) and for endogenous calnexin, by using α-CNX primary antibody and Alexa Fluor 568-labelled secondary antibody (c, g: red color). Nuclei are counter labelled with DAPI (a, e: blue color). Merged images (d, h) of the blue, green and red channels are shown in the last column. B, Live cell imaging of the subcellular localization of the control fluorescent proteins (a, d: green color) and calnexin (b, e: red color) in the stable-transgenic EYFP-FLAG (a-c) and EGFP (d-f) cells. To monitor calnexin, cells are transiently transfected by a plasmid encoding for mouse calnexin in fusion with a red fluorescent protein (OFPSpark, see Materials and Methods). Split images show two different field of view. Merged images from green and red channels are shown in the last column. Images are acquired with 100x oil immersion objective of a VisiScope CSU-W1 spinning disk confocal microscope. Scale bars: 10 μm.

Supplemental Figure S10

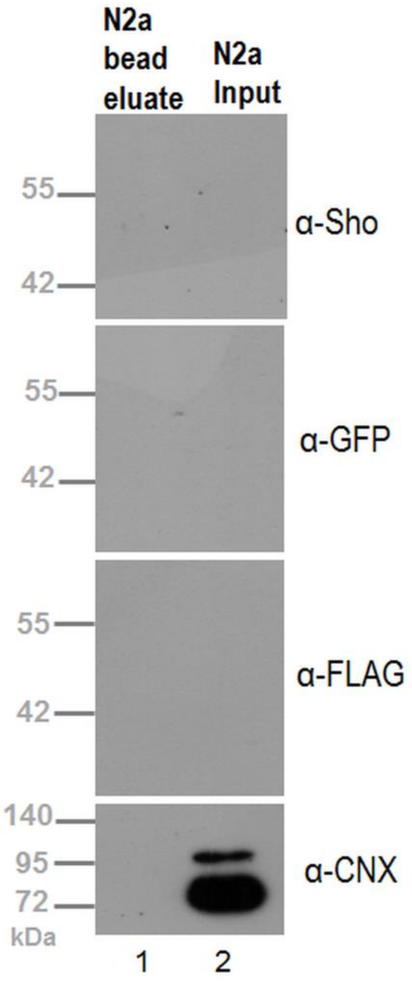

Supplemental Figure S10. Parental N2a cells probed as a negative control for co-immunoprecipitation of Shadoo and calnexin using anti-FLAG beads and total cell lysates. Bead pulled proteins (N2a bead eluate) in parallel with the input cell lysate (N2a input) are probed by Western blotting for Shadoo, (α-Sho), EYFP (α-GFP) and FLAG (α-FLAG) and for calnexin by α-CNX antibody. All gels used are 10% PA SDS gels, except for α-β-actin, which is 12% PA SDS gel.
